# 1356. Case Series of Children with Hepatitis and Adenovirus Infection, Alabama, October 2021—February 2022

**DOI:** 10.1093/ofid/ofac492.1185

**Published:** 2022-12-15

**Authors:** Markus A Buchfellner, L Amanda Ingram, Henry Shiau, Luz Helena Gutierrez Sanchez, Stephanie Saaybi, William Britt, Veronica Sanchez, Jennifer L Potter, David Kelly, Xiaoyan Lu, Stephanie Ayers-Millsap, Wesley G Willeford, Negar Rassaei, Hannah Bullock, Sarah Reagen-Steiner, Ali Martin, Daryl M Lamson, Umesh D Parashar, Aron J Hall, Adam MacNeil, Jacqueline E Tate, Hannah L Kirking

**Affiliations:** Division of Pediatric Infectious Diseases, University of Alabama at Birmingham, Birmingham, Alabama; Alabama Department of Public Health, Montgomery, Alabama; Division of Pediatric Gastroenterology, Hepatology and Nutrition, University of Alabama at Birmingham, Children's of Alabama, Birmingham, Alabama; Division of Pediatric Gastroenterology, Hepatology, and Nutrition, University of Alabama at Birmingham, Children's of Alabama, Birmingham, Alabama; Division of Pediatric Gastroenterology, Hepatology, and Nutrition, University of Alabama at Birmingham, Birmingham, Alabama; Division of Pediatric Infectious Diseases, University of Alabama at Birmingham, Birmingham, Alabama; Division of Pediatric Infectious Diseases, University of Alabama at Birmingham, Birmingham, Alabama; Division of Pediatric Infectious Diseases, University of Alabama at Birmingham, Birmingham, Alabama; Division of Pathology, University of Alabama at Birmingham, Children's of Alabama, Birmingham, Alabama; Centers for Disease Control and Prevention, Atalnta, Georgia; Jefferson County Department of Health, Birmingham, Alabama; Jefferson County Department of Health, Birmingham, Alabama; Division of High-Consequence Pathogens and Pathology, National Center for Emerging and Zoonotic Infectious Diseases, CDC, Atlanta, Georgia; Division of High-Consequence Pathogens and Pathology, National Center for Emerging and Zoonotic Infectious Diseases, CDC, Atlanta, Georgia; Division of High-Consequence Pathogens and Pathology, National Center for Emerging and Zoonotic Infectious Diseases, CDC, Atlanta, Georgia; Alabama Department of Public Health, Montgomery, Alabama; Wadsworth Center, New York State Department of Health, Albany, New York; Division of Viral Diseases, National Center for Immunization and Respiratory Diseases, CDC, Atlanta, Georgia; Division of Viral Diseases, National Center for Immunization and Respiratory Diseases, CDC, Atlanta, Georgia; Division of Viral Diseases, National Center for Immunization and Respiratory Diseases, CDC, Atlanta, Georgia; Division of Viral Diseases, National Center for Immunization and Respiratory Diseases, CDC, Atlanta, Georgia; Division of Viral Diseases, National Center for Immunization and Respiratory Diseases, CDC, Atlanta, Georgia

## Abstract

**Background:**

Human adenoviruses (HAdV) are a common cause of respiratory and gastrointestinal infections but rarely cause end-organ disease in children. From October 2021 to February 2022, several previously healthy children admitted to a single center for significant hepatitis also tested positive for HAdV. The aim of this investigation is to describe characteristics of these children.

**Methods:**

Children admitted to Children’s of Alabama (COA) from October 2021 to February 2022 with hepatitis who tested positive for HAdV by whole blood RT-PCR were included. Demographic, clinical, laboratory and treatment data were collected from medical records. Residual blood specimens were sent for adenovirus typing.

**Results:**

Nine pediatric patients with hepatitis and HAdV infection were identified (78% female; median age 3.0 years; IQR 1.7-3.0). Before admission, six reported diarrhea and three had respiratory symptoms. At presentation, eight had scleral icterus, six had jaundice, seven had hepatomegaly, and one was encephalopathic. All patients had elevated transaminases (AST range: 447-4000 U/L, ALT range: 784-4695 U/L); initial total bilirubin varied [range 0.23-13.5 mg/dL]). All had confirmed HAdV by RT-PCR on whole blood (initial qPCR range: 991-70,680 copies/mL). Notably, two children who transferred to another facility were negative for HAdV by RT-PCR when plasma was tested (instead of whole blood). Six children underwent liver biopsy showing varying degrees of hepatitis with no adenovirus detected on immunohistochemistry stains. Five children had HAdV type 41 confirmed. Three children presented or progressed to acute liver failure, two children were treated with cidofovir, and two underwent successful liver transplantation. No known epidemiologic links between patients were identified and all were from geographically distinct parts of Alabama.

**Conclusion:**

HAdV is a potentially underrecognized cause of hepatitis. Whole blood specimens may be preferred over plasma for HAdV RT-PCR testing. HAdV type 41 was identified in all patients with typing results available. Improved type-based surveillance may help determine HAdV patterns of circulation and inform future diagnostic testing.

**Disclosures:**

**William Britt, MD**, First Energy Corporation: Stocks/Bonds|Hookipa Pharma: Advisor/Consultant|Kroger Care: Stocks/Bonds|MDU Resources Group: Stocks/Bonds|PG&E Corporation: Stocks/Bonds.

